# Community curation in PomBase: enabling fission yeast experts to provide detailed, standardized, sharable annotation from research publications

**DOI:** 10.1093/database/baaa028

**Published:** 2020-04-30

**Authors:** Antonia Lock, Midori A Harris, Kim Rutherford, Jacqueline Hayles, Valerie Wood

**Affiliations:** 1 Department of Genetics, Evolution and Environment, University College London, Gower street, London WC1E 6BT, UK; 2 Department of Biochemistry, University of Cambridge, Tennis Court Road, Cambridge CB2 1GA, UK; 3 Cell Cycle Laboratory, The Francis Crick Institute, Midland Rd, London NW1 1AT, UK

## Abstract

Maximizing the impact and value of scientific research requires efficient knowledge distribution, which increasingly depends on the integration of standardized published data into online databases. To make data integration more comprehensive and efficient for fission yeast research, PomBase has pioneered a community curation effort that engages publication authors directly in FAIR-sharing of data representing detailed biological knowledge from hypothesis-driven experiments. Canto, an intuitive online curation tool that enables biologists to describe their detailed functional data using shared ontologies, forms the core of PomBase’s system. With 8 years’ experience, and as the author response rate reaches 50%, we review community curation progress and the insights we have gained from the project. We highlight incentives and nudges we deploy to maximize participation, and summarize project outcomes, which include increased knowledge integration and dissemination as well as the unanticipated added value arising from co-curation by publication authors and professional curators.

## Introduction

The key outputs of biological research—the data produced and the new knowledge gained—are primarily communicated via peer-reviewed publications. To increase the value of this knowledge, biocurators extract data from traditionally formatted publications in a suitably standardized way (1), which facilitates and accelerates data distribution to the research community via online databases, in line with FAIR (Findable, Accessible, Interoperable and Reusable) data sharing principles (2).

Biocuration adds value to published research by integrating information from different publications seamlessly and consistently. Biological knowledge can be organized and shared at the level of gene products or data types, as well as within and between species, and can be made interpretable by both humans and computers. Data also increases in value as the accuracy, detail, completion and connectivity of curation increase. Failure to integrate raw data into repositories, and new knowledge into curated resources, reduces data discoverability and re-use. Optimizing the impact of new and existing research, and obtaining maximal value from the initial investment, thus requires the expert data acquisition that biocuration provides (3).

## The big challenge of in-depth function curation

Large-scale datasets generated by high-throughput (HTP) methods typically contain data of one or a few homogenous types and can therefore be described and shared consistently with relative ease. Few HTP datasets, however, can be used in functional annotation of gene products (4). In-depth functional studies, whether follow-ups to HTP studies or standalone, pose a much greater challenge for accurate and consistent biocuration because they contain more complex, heterogeneous data. These low-throughput hypothesis-directed experiments are nevertheless critical for deriving new knowledge (5), such as novel gene characterizations or new mechanistic detail.

We recently showed that the numbers of both genes studied and different data types presented in ‘low-throughput’ publications have substantially increased in recent years (6). A well-characterized and well-annotated gene product in a model species may be associated with hundreds of publications, most of which provide some unique knowledge. Rapid access to this detailed gene-specific knowledge is fundamental to the biological research community, but it is prohibitively time-consuming and cost-ineffective for researchers to collate individually. Although submission of HTP datasets to repositories is generally mandated by funders and journals, currently there is no similar requirement for researchers to describe and submit knowledge gained from in-depth gene-specific publications to online resources.

Community curation has emerged as a promising approach to improve the dissemination of knowledge from in-depth publications and provide a cost-effective way to improve the accuracy and scalability of biocuration. Several biological databases include author contributions in their functional curation strategy to some extent (7–9). Notably, WormBase successfully solicits first-pass annotation from users and has pioneered integrating author curation with the publication process (9). FlyBase sends email requests to authors of new publications, inviting them to list the genes and data types described via an online tool (10) and has also mobilized the community to write gene summary paragraphs (11).

In July 2012, PomBase, the model organism database for the fission yeast *Schizosaccharomyces pombe*, launched a community curation program to enable author curation of in-depth molecular data. Here we report our approach to community curation, the contribution of community curators so far, and the nudges we employ to increase community participation. We highlight features that can be adopted by other groups considering community curation, and note the high quality of functional annotation produced by the effective collaboration between professional and community curators that has arisen in the PomBase system.

## The PomBase approach to community curation

### Community size and features

The fission yeast research community comprises approximately 2000 researchers publishing 500 papers a year. The community depends heavily on PomBase, with 81% reporting daily or weekly use (PomBase 2013 user survey; ftp://ftp.pombase.org/pombe/documents/2013_pombase_survey_summary.pdf), and consequently most researchers have become familiar with the data descriptors that databases use to describe their areas of interest. With its combination of a small database staff, but a highly engaged user community, PomBase was ideally placed to take an innovative approach to develop community curation as a way to sustain productivity. The key feature that distinguishes the PomBase approach from other community curation efforts is that we aim to enable authors to fully curate a publication using detailed ontology terms for a wide range of data types to the same standard as a professional biocurator.

**Figure 1:**
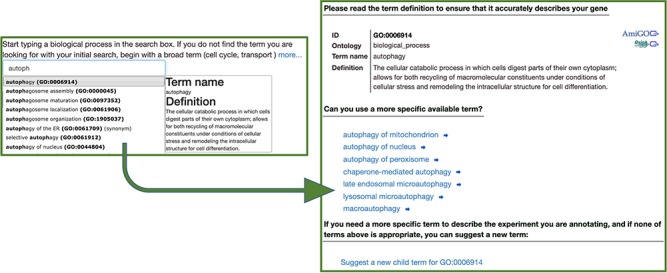
The Canto annotation tool. A user-friendly step-by-step annotation workflow guides new users through finding ontology terms and completing annotations.

### Curation tool, literature management and curation

The open source web-based curation tool Canto (https://curation.pombase.org/) was developed by PomBase to support curation and literature management by professional curators and the community (12). Fission yeast publication details (authors, abstract, date, journal details) are retrieved from PubMed daily by a pipeline configured to use keywords for the domain of interest (for PomBase, the scientific and common organism names). Curation staff manually classify publications as ‘curatable’, ‘HTP’, ‘review’, ‘methods’ and ‘wrong species’ based on the abstract. Curatable publications are those where the abstract indicates that they are likely to contain gene-specific molecular data (biochemical or genetic) from focused, hypothesis-driven experiments. The corresponding author details (name and e-mail) are retrieved from Canto (or entered into Canto for new researchers), and associated with curatable manuscripts. Configurable templates are used to generate personally addressed invitations that contain basic curation instructions and a link to the curation session and are e-mailed to the corresponding author from Canto. Corresponding authors may choose to ‘re-assign’ sessions to a co-author within Canto. Curation status is updated in the Canto administrative interface: publications are automatically recorded as either ‘unassigned’, ‘assigned to a curator’, ‘active’ (session accepted, curation in progress, curation paused), ‘submitted for approval’ or ‘approved’ depending on activity.

The Canto interface guides authors through steps to list the genes studied in a publication and then to add annotations based on experimental results in the paper. The workflow depends largely on the type and number of annotations that can be made from the data in the paper. Canto provides extensive online documentation describing annotation workflows for various data types, and all can be explored further using the Canto demo server (https://curation.pombase.org/demo/). For PomBase, Canto supports ontology-based annotations, including all three branches of the Gene Ontology (GO) (13,14), phenotypes using the Fission Yeast Phenotype Ontology (FYPO) (15) and protein modifications using PSI-MOD (16). Genetic and physical interactions are captured using the BioGRID system (17). For ontology annotations, the Canto interface helps users find and use the most specific terms possible to describe observations (Figure 1). Canto also gathers supporting details including annotation provenance (using the Evidence and Conclusion Ontology (ECO) (18)), interacting genes and (for phenotypes) alleles, genotypes and conditions. Applicable annotation extensions (19) such as enzyme substrates, connections between Gene Ontology aspects and phenotype severity and penetrance, are collected via context-specific and biologist-friendly prompts (for example, Canto prompts for ‘substrates’ of enzymes rather than ‘inputs’ and avoids or explains technical jargon such as ‘annotation extensions’ in the workflow).

### Response rate and metrics

**Figure 2:**
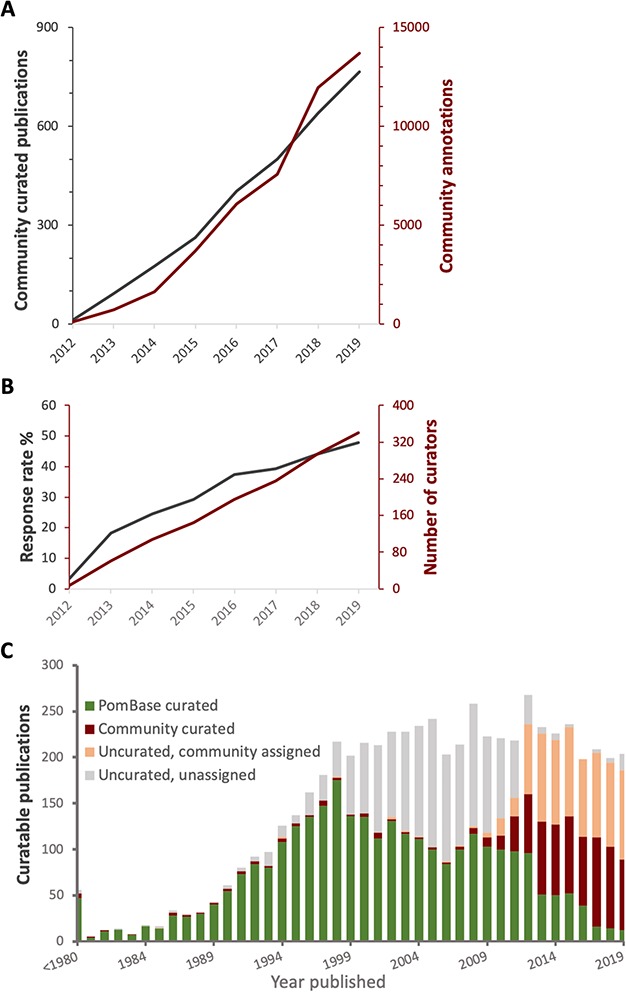
Curation statistics. **A**. Cumulative numbers of publications curated (grey) and annotations added (red) bythe community over time. **B**. Cumulative number of new participants (red) and invitation response rate (grey) over time. **C**. The proportion of curatable fission yeast literature curated by PomBase staff and community, or as yet uncurated, by year published. Uncurated publications are subdivided into ‘uncurated community’ (invitation sent, no response) and ‘uncurated unassigned’ (no invitation sent—we do not send invitations for publications predating 2012).

To date (February 2019), a total of 789 low-throughput publications have been curated either fully or partially by the fission yeast community using Canto, providing 13 982 highly specific annotations (Figure 2A). The overall response rate has risen from 18% in 2013 to 50%, and the number of participating authors is steadily increasing (Figure 2B). Although curation is only solicited for papers published since 2012, community members have curated more than 130 earlier publications, some dating as far back as the 1970s (Figure 2C).

### Increasing participation

Because publication curation is not mandated by funders or journals, we need to identify alternative ways to increase the participation rate. We turned to ‘nudge theory’ (20), a set of approaches based on the behavioural science proposition that individual decision-making can be influenced by multiple small positive changes. In the UK this technique has been adopted by the government to guide policy and regulation (21) and has led to particular successful results in the health sciences (22). Specific aspects of the PomBase community curation project fit the four broad principles described by the Behavioural Insights Team (https://www.bi.team/wp-content/uploads/2015/07/BIT-Publication-EAST_FA_WEB.pdf), whereby making an activity Easy, Attractive, Social and Timely (EAST) increases participation.

**Figure 3:**
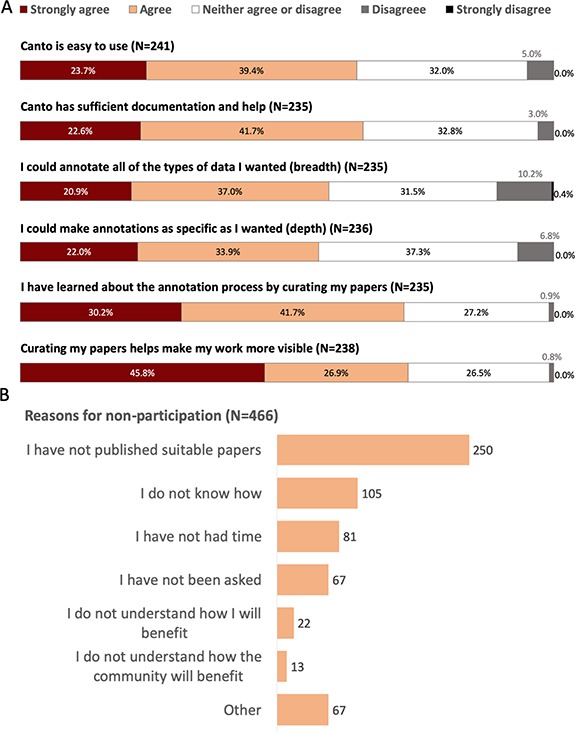
Community perception of Canto curation from 2019 PomBase user survey. **A**. Responses to questions evaluating Canto usability. Note: 30% of respondents (189/632) reported having used Canto. **B**. Reasons given for not participating in community curation. Free text responses from respondents selecting ‘other’ indicate that many intend to participate in the future, or found that their papers had already been curated by other lab members.

### EAST: Easy

**Figure 4:**
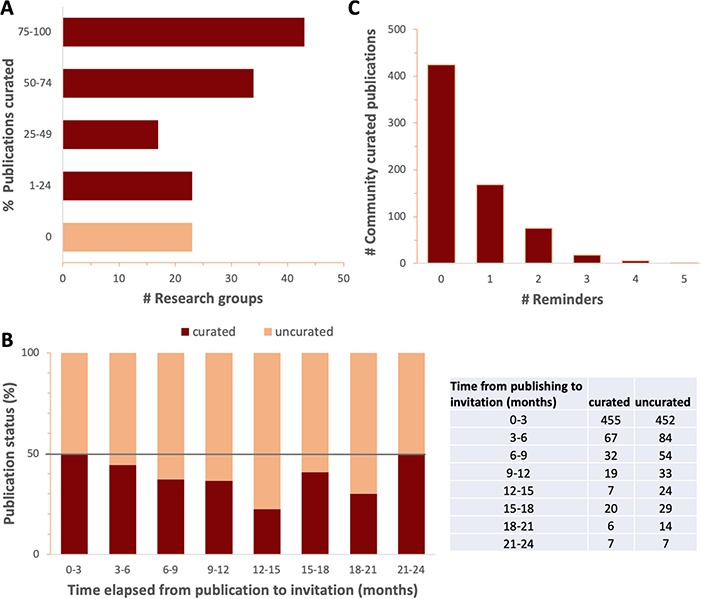
Response to curation invitations. **A**. Percent of publications curated per research group for all groups that have received four or more curation invitations. Research groups with fewer than 4 relevant publications were omitted to focus on research groups dedicated to fission yeast. **B**. Percent (graph) and number (table) of curated and uncurated publications as a function of the time elapsed between PubMed indexing and the date that the first invitation to curate was sent, in 3-month intervals. The increase in response rate seen in later time intervals could either reflect the increased number of reminders received, or simply be an artefact of the smaller sample sizes. **C**. The number of community curated publications versus the number of reminders.

Because we could not assume that publication authors in the PomBase user community had prior knowledge of bio-ontologies and their structure, or the resources and standards used to describe knowledge, it was essential to design Canto with intuitive annotation interfaces. The biologist-friendly workflows described above are supplemented by comprehensive in-line tooltips, and full documentation available from each page. In our 2019 user survey (ftp://ftp.pombase.org/pombe/documents/2019_pombase_survey_summary_no_freetext_responses.pdf), 63% of Canto users agreed or strongly agreed that the tool is easy to use, and 64% agreed that sufficient help is available (Figure 3A). We also continually seek user feedback, and monitor mistakes or omissions in completed curation sessions, to identify ways we can improve Canto’s features, behaviour and documentation. Once a research group participates in curation, they are likely to continue to do so, and to curate most of their publications, indicating that the dropout rate is low, and the curation process is accessible (Figure 4A).

#### Improvements to the curation process

One of the main challenges in making the curation process accessible to non-experts is to facilitate navigation of the very large bio-ontologies used to describe data. For example, GO contains more than 40 000 terms and FYPO over 7000. To simplify term selection, PomBase Canto now excludes nearly 15 000 GO terms that are not applicable to fission yeast due to taxonomic specificity. Ontology searching is also facilitated by ensuring that terms commonly used by the community are available as ontology term names or synonyms.

#### Continuous improvements to the Canto curation tool workflow

If specific curation problems recur even after documentation improvements, the Canto interface itself may require changes. Canto’s modular organization and configuration makes testing the effect of minor interface or text changes (e.g. rearranging buttons or table layouts, adding new prompts, or reorganizing actions) straightforward. More serious workflow issues may require more extensive changes, but the Canto code organization allows even these to be implemented quickly. Additionally, as community curators become more proficient at curation, they may prefer to use the advanced options and shortcuts available to professional curators.

### EAST: Attractive

We have identified numerous benefits that participants derive from community curation and strive to promote these benefits to prospective contributors.

#### Increased dissemination

Most funding agencies now require grant holders to produce data management plans that demonstrate the ways they disseminate the data produced by the funded work. Community curation provides a novel opportunity to satisfy this requirement. To support this use, PomBase publication pages display curator attribution. We also record contributors’ ORCIDs (https://orcid.org/) and are exploring ways to make user curation records available in a format suitable for grant reporting. Seven principal investigators have already reported community curation as a data management or data dissemination mechanism in grant proposals, and 22 propose to do so in the immediate future (pers. comm.).

#### Increased visibility

To realize the full potential value of research results, data must be findable and readily reusable. The traceable dataset accession numbers that are increasingly cited for ‘findability’, though necessary, are not sufficient. First, their use depends on prior knowledge of specific studies; second, and more importantly, functional curation is essential to make small-scale data as findable as HTP data. The curation process attaches many attributes to gene products, such as ontology term IDs and supporting references, that make functional data more findable. Most fission yeast publication authors agree that curation makes their research more visible (73% of 2019 user survey respondents agree or strongly agree; Figure 3A). Furthermore, since PomBase staff focus their curation efforts on papers published before 2014, community curation is an indispensable route to database inclusion and subsequent dissemination for new publications.

#### Increased database understanding and data reuse by participants

Researchers who understand the curation process and how data is described and integrated in databases can make more efficient use of curated data in research activities. For example, understanding how annotation is inferred via ontology relationships allows biologists to devise database search strategies, and to optimize data analyses such as GO term enrichment. In our user survey 72% of respondents who had participated in curation reported an increased understanding of the curation process (Figure 3A). Encouragingly, the most common reason reported for non-participation was not having published suitable papers, and very few respondents chose options indicating that they did not understand how they or the community would benefit from community curation (Figure 3B).

### EAST: Social

Social norms and attitudes influence our choices and we are more likely to engage in a behaviour when we believe that those we identify with engage in the behaviour (23). Accordingly, we regard promoting a sense of community, and establishing community curation as a routine task performed by everyone as part of the publication process, as critical for PomBase community curation to thrive.

Canto associates each publication and curation session with a corresponding author, enabling us to use personally addressed emails to provide social incentives to participate in curation. We believe that invitation emails addressed directly to the participant by name, and signed off by an individual PomBase curator, are much less likely to be ignored than an impersonal message that appears auto-generated. We frequently receive replies stating when a participant will be available to submit data, and even sometimes apologizing for delays.

To promote curation as a community activity the PomBase home page features a prominent panel linking to publications recently curated by their authors. We maintain metrics of curation progress (https://curation.pombase.org/pombe/stats/annotation), which identify the growing community contribution to the overall PomBase annotation corpus (percentages, numbers of publications, numbers of annotations). Graphical abstracts from selected curation sessions are showcased in our front page ‘Research spotlight’ panel. Social media also plays an increasingly important role in raising awareness about community curation and in raising the visibility of community curated publications. All PomBase pages link to the PomBase twitter feed (@PomBase), which is frequently used to highlight newly incorporated data.

#### Effective co-curation

Once a curation session is submitted, annotations are then checked by a professional curator who often extends or refines the curation in consultation with the authors. The author will often suggest further improvements at this point. This iterative process takes full advantage of the professional curator’s knowledge of bio-ontologies and best annotation practices, coupled to the author’s expert knowledge of the biology.

### EAST: Timely

Participation in an activity depends critically on prompting people when they are most receptive. Publications are increasingly curated by the first author, often PhD students or post-docs who are transiently associated with a laboratory, and may be less likely to participate after they move to a new institution or different field. Figure 4B shows that participation is highest among authors contacted within 3 months after publication, suggesting that immediacy is key to a high response rate. Our current model is to send invitations for all new curatable papers within a few days of PubMed indexing. Once a researcher has submitted curation for a new publication, we often invite them to curate their older papers, because having the procedures fresh in mind makes curating additional papers easier.

Because some authors are unable to commit time immediately, we send reminders at semi-regular intervals on the order of every few months. Although many authors curate on the first request in the first year, in some cases, papers have been curated after four or five reminders (Figure 4C). Figure 2C shows the number of papers curated by publication year, and those still assigned to the community for curation. Currently we do not intervene to curate any of the papers assigned for community curation because we are aware that many laboratories who have not yet participated still intend to do so (Figure 3B). Furthermore, sending reminders always brings in batches of new publications that fill our capacity for checking, so to date we have not needed to prioritize non-curated papers.

In early 2017, the PomBase website was redesigned to support daily updates (as opposed to quarterly) (24), allowing curated data to become visible on the website promptly, usually within a week of submission. The new website also features community curation on the home page. We observed a marked increase in participation rate between 2017 and 2018 (Figure 2A), which we attribute at least in part to the more rapid turnaround from publication, to curation submission, to data hosting in PomBase that became possible at that time.

### Lessons learned and future directions

Since launching the PomBase community curation project, we have shown that enhancing the curation procedure, promoting its benefits, engaging the community and making timely requests have improved the participation over the past 6 years, such that community curation is now an established practice in the fission yeast community. Our experience shows that community curation provides a robust route to capturing biological research at scale and thus represents a valuable supplement to professional curation as we contend with ever-increasing amounts of published data and static or declining database resource support.

The accuracy of community curation is very high and changes (as opposed to additions) to submitted curation are rare. We have identified only a few examples in the past several months where annotations were revised by the checker. In one case, the author used a ‘cellular response to hypoxia’ phenotype annotation for data that we would record as expression level changes (the latter data type is not yet available in Canto, but can be added by curators). In the second example, an author annotated a ‘resistance to chemical’ phenotype because one mutant grew better than another. Compared to wild type, however, growth was normal, and PomBase phenotypes all use wild type as the reference. Usually, community curator errors resemble those made by an inexperienced curator, such as a recent example in which a ‘cellular response to’ GO biological process term was revised to phenotype annotations. Many such errors reflect obscure details of curation practice rather than biological interpretation. Reciprocally, annotation refinements provided by curators are approved by the authors, who often spot errors made by professional curators such as forgetting to change evidence, conditions or extensions after using the copy-edit feature, or connecting the wrong mutant allele to a phenotype. Occasionally the community will ask us to remove curation they believe is unimportant or misleading. The community also provide useful feedback on the accuracy of GO terms in their area of expertise. Overall, we see no qualitative difference in accuracy between authors and professional curators, but community curators are currently much less likely to provide comprehensive annotation.

Community curation thus does more than simply increase curation speed. The dialogue that the curation-and-review cycle establishes between the professional and the community curator, in which authors often suggest further improvements, yields higher quality annotations than either individual would produce alone. The Reactome co-curation and review model (7) also illustrates this phenomenon. In effect, we have developed an iterative process that takes full advantage of the professional curator’s knowledge of bio-ontologies and best annotation practices, coupled to the author’s expert knowledge of the biology. This synergistic effect counters any concern that community curation merely redistributes costs from professional curators to authors instead of improving value for money (25). Once the benefits that we observe, including high annotation quality, widespread understanding of curation-related concepts, tools and practices, and—perhaps most important—increased visibility and reuse of data and knowledge, are factored in, the view of author–curator collaboration becomes far more optimistic.

One confounding factor in evaluating the individual methods we employ to maximize participation in community curation is that changes are made incrementally in parallel, and with no control group. Nevertheless, the steadily increasing uptake over time (from 18% at the end of 2013 to 50% today), the various qualitative survey responses, and analyses of response rates over time give us confidence that, taken together, the methods we have implemented have all contributed to improved participation rates. We will continue to explore and test additional mechanisms to attract more participants. In the near future we plan to introduce microPublications (26,27), which will include curation as an integral part of publishing, and enhance Canto curation of ‘traditional’ publications by using ORCID identifiers (7) to manage contributions. We will also produce instructional videos to supplement existing documentation and provide directed one-to-one tutorials by Skype for non-participating laboratories. Universal uptake by the entire research community (our ultimate goal) will likely only be achieved if curation is made a mandatory condition of publication or funding.

Over the long term, community curation provides a viable option for improving curator productivity, curation accuracy, data dissemination and findability. Community curation could also offer a route for other resources, especially emerging model species without dedicated curation staff, to acquire high-quality functional annotation that can be readily integrated with data available for more thoroughly studied species. A number of communities have already expressed interest in this route (28). A wide range of research communities, and the scientific efforts they pursue, thus stand to benefit from widening participation in literature curation.
